# A Dynamical Phyllotaxis Model to Determine Floral Organ Number

**DOI:** 10.1371/journal.pcbi.1004145

**Published:** 2015-05-07

**Authors:** Miho S. Kitazawa, Koichi Fujimoto

**Affiliations:** 1 Department of Biological Sciences, Osaka University, Toyonaka, Osaka, Japan; 2 Japan Society for the Promotion of Science, Tokyo, Japan; University Paris Diderot, FRANCE

## Abstract

How organisms determine particular organ numbers is a fundamental key to the development of precise body structures; however, the developmental mechanisms underlying organ-number determination are unclear. In many eudicot plants, the primordia of sepals and petals (the floral organs) first arise sequentially at the edge of a circular, undifferentiated region called the floral meristem, and later transition into a concentric arrangement called a whorl, which includes four or five organs. The properties controlling the transition to whorls comprising particular numbers of organs is little explored. We propose a development-based model of floral organ-number determination, improving upon earlier models of plant phyllotaxis that assumed two developmental processes: the sequential initiation of primordia in the least crowded space around the meristem and the constant growth of the tip of the stem. By introducing mutual repulsion among primordia into the growth process, we numerically and analytically show that the whorled arrangement emerges spontaneously from the sequential initiation of primordia. Moreover, by allowing the strength of the inhibition exerted by each primordium to decrease as the primordium ages, we show that pentamerous whorls, in which the angular and radial positions of the primordia are consistent with those observed in sepal and petal primordia in *Silene coeli-rosa*, Caryophyllaceae, become the dominant arrangement. The organ number within the outmost whorl, corresponding to the sepals, takes a value of four or five in a much wider parameter space than that in which it takes a value of six or seven. These results suggest that mutual repulsion among primordia during growth and a temporal decrease in the strength of the inhibition during initiation are required for the development of the tetramerous and pentamerous whorls common in eudicots.

## Author Summary

Why do most eudicot flowers have either four or five petals? This fundamental and attractive problem in botany has been little investigated. Here, we identify the properties responsible for organ-number determination in floral development using mathematical modeling. Earlier experimental and theoretical studies showed that the arrangements of preexisting organs determine where a new organ will arise. Expanding upon those studies, we integrated two interactions between floral organs: (1) spatially and temporally decreased inhibition of new organ initiation by preexisting organs, and (2) mutual repulsion among organs such that they are “pushed around” during floral development. In computer simulations incorporating such initiation inhibition and mutual repulsion, the floral organs spontaneously formed several circles, consistent with the concentric circular arrangement of sepals and petals in eudicot flowers. Each circle tended to contain four or five organs arranged in positions that agreed quantitatively with the organ positions in the pentamerous flower, *Silene coeli-rosa*, Caryophyllaceae. These results suggest that the temporal decay of initiation inhibition and the mutual repulsion among growing organs determine the particular organ number during eudicot floral development.

## Introduction

How to determine the numbers of body parts is a fundamental problem for the development of complete body structures in multicellular organisms. Digit numbers in vertebrates are evolutionarily optimized for the specific demands of the organism [1]; the body-segment number in insects is constant despite the evolutionarily diversified gene regulation in each segment [2–4]; and five petals are indispensable to forming the butterfly-like shape that is unique to legume flowers [5]. Studies of animal structures, such as vertebrate limbs and insect segments, strongly suggest that crosstalk between pre-patterns (e.g., morphogen gradients) and self-organizing patterns underlies the developmental process of organ-number determination [6–13]. In plant development, a self-organization based on the polar transport of the phytohormone auxin [14–16] is conserved among seed plants [17] and seems to be the main regulator of the development of a hierarchal body plan, called a shoot, consisting of a stem and lateral organs such as leaves. The number of concentration peaks in most self-organizing patterns, such as Turing pattern and the mechanisms proposed for plant-pattern formation, is proportional to the field size [15, 18, 19]. Despite having a diversified field size for floral-organ patterning, the eudicots, the most diverged clade among plants, commonly have pentamerous or tetramerous flowers containing five or four sepals and petals (the outer floral organs), respectively, and rarely have other numbers of organs [20, 21]. Here, we focus on the developmental properties that so precisely and universally determine the floral organ numbers through self-organizing processes.

Phyllotaxis, the arrangement of leaves around the stem, provides insight into floral development, because studies of floral organ-identity determination [22] have verified Goethe’s foliar theory, which insists that a flower is a short shoot with specialized leaves [23]. Phyllotaxis is mainly classified into two types: spiral phyllotaxis, which has a constant divergence angle and internode length, and whorled phyllotaxis, which has several leaves at the same level of a stem [24]. For spiral phyllotaxis, Hofmeister described a hypothesis of pattern formation in 1868 [24], which we summarize in three basic rules: the time periodicity of primordia initiation, the initiation of a primordium at the largest available space at the edge of the meristem (the undifferentiated stem-cell region), and the relative movement of primordia in a centrifugal direction from the apex due to the growth of the stem tip. Following that hypothesis, numerous mathematical models incorporating contact pressure [25, 26], inhibitor diffusion [27], reaction-diffusion [18, 28], and mechanical buckling of the epidermis [29, 30] were proposed to explain the observed phyllotactic patterns. Over the past ten years, these mathematical models were tested and interpreted in light of modern molecular biology. Several studies have suggested that the competitive polar transport of the auxin accounts for two of Hofmeister’s rules, the periodicity of initiation and the initiation at the largest space, which together are capable of reproducing both spiral phyllotaxis and whorled phyllotaxis [15, 16, 31].

Despite their simple rules and uncertain molecular basis, the phyllotaxis models can account for several of the quantitative properties observed in organ patterning. For example, one model showed that the divergence angle between successive leaves is 180 degrees for the first and second leaves, 90 degrees for the second and third leaves, and oscillating thereafter, converging to the golden angle, 137.5 degrees, which agrees with the phyllotaxis of true leaves in *Arabidopsis thaliana* after the two cotyledons [32, 33]. Similar oscillatory convergence to a particular divergence angle occurs in the sepal primordia of the pentamerous flower of *Silene coeli-rosa*, Caryophyllaceae. In *S*. *coeli-rosa*, the divergence angle is 156 degrees at first, and then it oscillates, converging on 144 degrees [34]. The golden angle also appears in the floral organs of several Ranunculaceae species [35, 36]. The agreements between the phyllotaxis models and actual floral development suggest that mathematical models can give useful clues to the underlying mechanisms of not only phyllotaxis but also floral organ patterning.

**Fig 1:**
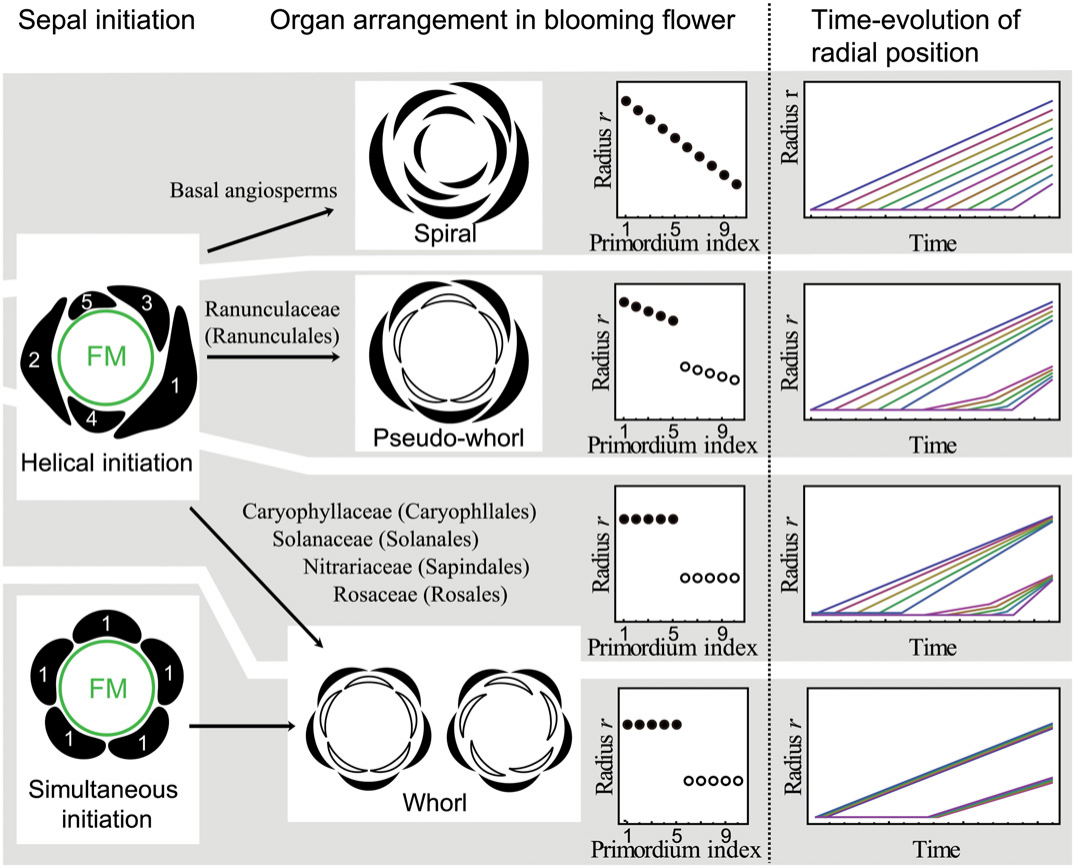
Schematic diagram for pentamerous flower development. Sepal initiation (the first row), arrangement of sepal (black) and petal (white) whorls in blooming flower (the second row). Green circle represents a floral meristem (FM). Index numbers indicate the initiation order of five sepals. The radial position of the organs (the third row), namely the distance between the organ and floral apex, is spaced regularly in a spiral arrangement, whereas it has a gap between the fifth and sixth organs in the pentamerous pseudo-whorled and whorled arrangement. Regarding the hypothetical time evolution of the radial position (the fourth row), in all arrangements, the radial position increases with the progression of floral development. In the spiral arrangement, the radial position of the organ is always spaced regularly. In the pseudo-whorled and whorled arrangement subsequent to helical initiation, the radial position of organs within a whorl becomes closer during growth. In the whorled arrangement following simultaneous initiation, the radial position of the organs within a whorl is always identical.

There are at least three fundamental differences, however, between real floral development and the phyllotaxis models. The first difference is the assumption of constant primordium displacement during tip growth, which comes from Hofmeister’s hypothesis and has been incorporated into most phyllotaxis models. Although the helical initiation has been thought to always result in spiral phyllotaxis, many eudicots form the whorled-type sepal arrangements in their blooming flowers subsequent to helical initiation [37] (Fig 1; e.g., Caryophyllaceae [34], Solanaceae [38], Nitrariaceae [39], and Rosaceae [40]). The remnants of helical initiation are more obvious in the pseudo-whorls (e.g., Ranunculaceae [41]), where the distance between each organ primordium and the floral center varies slightly even in the whorls of mature flowers, which usually have more varied floral organ numbers [20, 35], suggesting that post-meristematic modifications of primordia positions [42] play an essential role in generating the whorled arrangement and determining the floral organ number during floral development. In contrast, most phyllotaxis models have assumed constant growth of the primordia, so that the whorls appear only after the simultaneous initiation of several primordia [19]. The second difference comes from the fact that floral development is a transient process, whereas most phyllotaxis models have focused on the steady state of the divergence angle. Although the golden angle (137.5 degrees) is quite close to the inner angle of regular pentagon (144 degrees), the developmental convergence from 180 degrees (cotyledon) to 137–144 degrees in phyllotaxis requires the initiation of more than five primordia, both in *A*. *thaliana* leaves and in the mathematical models [16, 33]. In contrast, the divergence angle between the second and third sepal primordia in pentamerous eudicot flower development is already close to 144 degrees [34]. The third difference comes from the accuracy of the floral organ number in many eudicots. Although the polar auxin-transport model reproduced both wild-type and mutant *A*. *thaliana* floral organ positioning [43], the organ number in the model was more variable, even with an identical parameter set (Fig 3 in [43]), than that in experimental observations (Table 1 in [44]). Moreover, among eudicot species, the appearance of pentamerous flowers is robust, despite the diversity of the meristem size and the outer structures, including the number and position of outside organs such as bracts [20]. Together, the differences between real floral development and previous phyllotaxis models indicate that floral development requires additional mechanisms to determine the particular organ number.

To resolve the inconsistencies between the earlier models and actual floral development, we set out a simple modeling framework, integrating Hofmeister’s rules with two additional assumptions, namely, the repulsion between primordia that can repress primordium growth and the temporal decrease in initiation inhibition of new primordium, which were proposed independently in the contact pressure model [25, 45, 46] and the inhibitory field model [33, 47, 48], respectively, for phyllotaxis. First, when we incorporated mutual repulsion among primordia into the growth process, a whorled-type pattern emerged spontaneously following the sequential initiation of primordia. The mutual repulsion obstructed the radial movement of a new primordium after a specific number of primordia arose, causing a new whorl to emerge. The number of primordia in the first whorl tended to be four or eight. Second, when we assumed that older primordia have less influence on the initiation of a new primordium, the pentamerous whorl arrangement, which is the most common arrangement in eudicot flowers, became dominant. We analytically show the conditions for the development of tetramerous and pentamerous whorls, and we predict possible molecular and physiological underpinnings.

## Model

Following Hofmeister’s rules as mathematically interpreted by Douady and Couder [49], we focused on initiation and growth, the two processes of floral development. In the initiation process, each primordium emerges successively at the least crowded position, depending on a potential function [49]. We assumed periodic initiation to examine how the sequential initiation results in the whorled-type pattern. We allowed the primordia to move during the growth process in response to the repulsion among the primordia, unlike earlier studies that assumed constant growth depending only on the distance from the apex [28, 49].

### The initiation process

**Fig 2:**
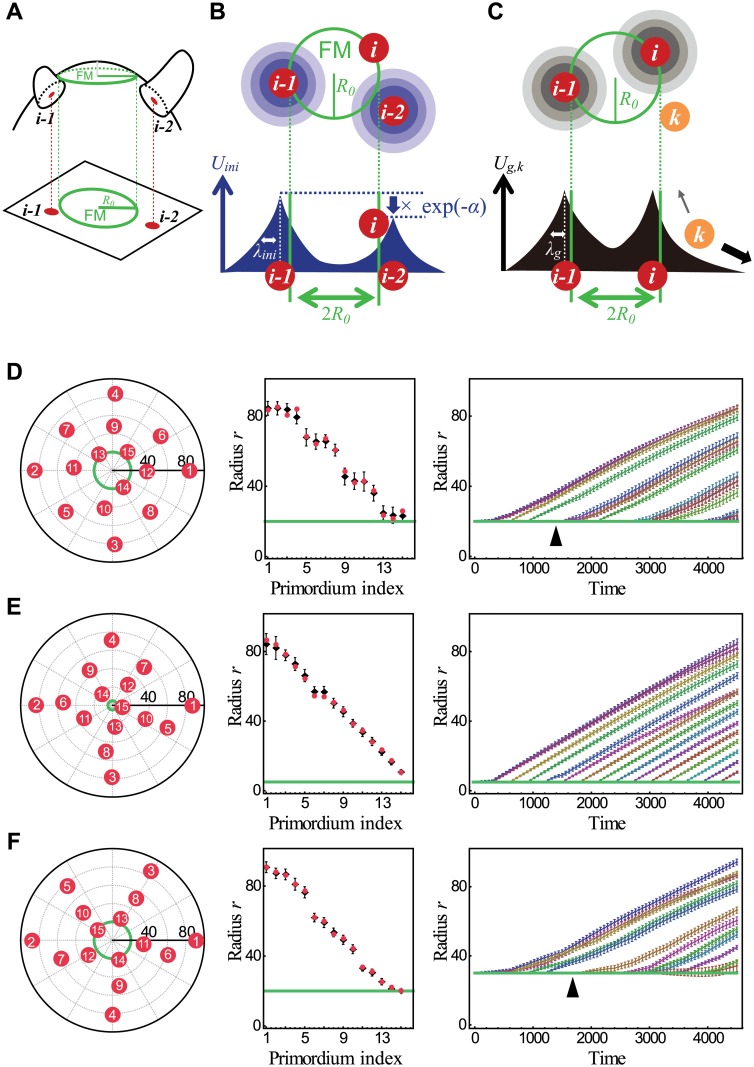
Emergence of multiple whorls in model simulations. **A.** Geometric assumptions of the model. **B.** The initiation process. A new primordium (*i*) is initiated at the edge of the floral meristem (FM; green circle) where the initiation potential *U*_*ini*_ takes the minimum value. *i*, *i* −1, and *i* −2 are the primordium indices that denote the initiation order. *U*_*ini*_ exponentially decreases with time (*α*) and the distance between primordia (*λ*_*ini*_). **C.** The growth process. Each primordium (*k*) moves at the outside of the circular FM, depending on the growth potential *U*_*g*, *k*_. Primordium *k* rarely moves against the gradient (grey thin arrow), but mostly follows the gradient (black thick arrow; see the Model section). **D–F.** Emergence of whorled-type pattern with increasing meristem radius *R*_0_ and temporal decay rate *α*. Left panels: Spatial pattern after 15 primordia (red circles) initiated in an indexed order at the meristem edge (green circle; *r* = *R*_0_). Middle panels: Radial distance (black) from the meristem center as a function of the primordium initiation index (left panel) averaged over 400 replicate Monte Carlo simulations. Error bars represent twice the S.D. Red circles are a set of representative samples. Right panels: Time evolution of the radial coordinates of each primordium averaged over 400 replicates. Error bars show 2 S.D. The arrowheads in **D** and **F** indicate the growth arrest of the fifth and sixth primordia, respectively. Colors denote the index of the primordia. Green line in the left, middle and right panels denotes the meristem edge. (*R*_0_, *α*) = (20.0,0.0) in **D**, (5.0,0.0) in **E** and (20.0,2.0) in **F**. *β* = 1.0 × 10^4^, *λ*_*ini*_ = *λ*_*g*_ = 10.0, *τ* = 300, and *σ*_*r*_ = *σ*_*θ*_ = 0.05 in **D**–**F**.

Following the earlier models [49], we represented the meristem as a circular disc with radius *R*_0_ and the primordia as points (Fig 2A). A new primordium arises at the point along the edge of the meristem (*R*_0_, *θ*), in polar coordinate with the origin at the meristem center, where *θ* gives the minimum value of the inhibition potential *U*_*ini*_. As one of the simplest setups for sequential initiation [37], we followed the assumption of earlier models for spiral phyllotaxis [49], which state that new primordia arise sequentially with time intervals *τ*, as opposed to the simultaneous initiation studied previously for whorled phyllotaxis [19] (Fig 1). Although the structures outside of the flower, such as bracts and other flowers, as well as the position of the inflorescence axis, may affect the position of organ primordia, the pentamerous whorls appear despite their various arrangement [20]. Therefore, as the first step of modelling of floral organ arrangement, we assumed that whorl formation is independent of any positional information from structures outside of the flower. Thus, we calculated the inhibition potential only from floral organ primordia which are derived from a single floral meristem.

The potential functions for the initiation inhibition by preexisting primordia have been extensively analyzed in phyllotaxis models [16, 47, 49]. The potential decreases with increasing distance between an initiating primordium and the preexisting primordia account for the diffusion of inhibitors secreted by the preexisting primordia [27, 50], and the polar auxin transport in the epidermal layer, as proposed in previous models of phyllotaxis [15, 16, 31] and the flowers [43]. We employed an exponential function exp(−*d*_*ij*_/*λ*_*ini*_) as a function of *θ*, where *d*_*ij*_ denotes the distance between a new primordium *i* and a preexisting primordium *j* at (*r*_*j*_, *θ*_*j*_) as

$$\begin{array}{c} d_{ij}=\sqrt{R_{0}^{2}+r_{j}^{2}-2R_{0}r_{j}\cos\left(\theta -\theta_{j}\right)}\cdot \end{array}$$ (1)

The function decreases spatially through the decay length *λ*_*ini*_ exponentially, induced by a mechanism proposed for the polar auxin transport, i.e., the up-the-gradient model [15, 16]. Up-the-gradient positive feedback amplifies local auxin concentration maxima and depletes auxin from the surrounding epidermis, causing spatially periodic concentration peaks to self-organize [15, 16] and thus determine the initiation position of the primordia [51]. The amplification and depletion work as short-range activation and long-range inhibition, respectively [52], which are common to Turing patterns of reaction-diffusion systems [18]. Since the interaction of local maxima in the reaction-diffusion systems follows the exponential potential [53, 54], the up-the-gradient model likely explains the exponential potential between the auxin maxima, while the rigorous derivation requires further research. The decay length *λ*_*ini*_ depends not only on the ratio of the auxin diffusion constant and the polar auxin-transport rate [15] but also on other biochemical parameters for polar transport and the underlying intracellular PIN1 cycling [55]. Another mechanism, referred to as the with-the-flux model [56, 57], has been proposed for the polar auxin transport. Although with-the-flux positive feedback can also produce spatial periodicity, the primordia position corresponds to auxin minima [57], which is inconsistent with observations [51].

On the other hand, the with-the-flux mechanism can explain auxin drain from the epidermal layer of the primordia to internal tissue [58]. Since the drain gets stronger as the primordia mature [58, 59], the auxin drain could cause decay of the potential depending on the primordia age. The auxin decrease in maturing organs can also be caused by controlling auxin biosynthesis [60, 61]. Therefore, we integrated another assumption, namely that the inhibition potential decreases exponentially with the primordia age at the decay rate *α* (Fig 2B). Temporally decaying inhibition was proposed previously to represent the degradation of some inhibitors [47, 48] and account for various types of phyllotaxis by simple extension of the inhibitory field model [33]. Taken together, the potential at the initiation of the *i*-th primordium is given by

$$\begin{array}{c} U_{ini}\left(\theta \right)=\sum_{j=1}^{i-1}\exp(-\alpha (i-j-1))\exp(-\frac{d_{ij}}{\lambda_{ini}})\cdot \end{array}$$ (2)

### The growth process

Most phyllotaxis models have assumed, based on Hofmeister’s hypothesis, that the primordia move outward at a constant radial drift depending only on the distance from the floral center without angular displacement, which makes helical initiation result in spiral phyllotaxis [49]. Here, we assumed instead that all primordia repel each other, even after the initiation, except for movement into the meristematic zone (Fig 2C) following observation of the absence of auxin (*DR5* expression) maxima at the center of the floral bud (e.g., [62]). Even at the peripheral zone away from the meristem, the growth is not limited. Hence there is no upper limit for the distance between primordia and the center. The repulsion exerted on the *k*-th primordium is represented by another exponentially decaying potential when there are *i* primordia (1 ≦ *k* ≦ *i*):

$$\begin{array}{c} U_{g,k}\left(r,\theta \right)=\sum_{j=1,j\neq k}^{i}\exp(-\frac{d_{kj}}{\lambda_{g}}), \end{array}$$ (3)

where the decay length, introduced as *λ*_*g*_, can differ from *λ*_*ini*_. The primordia descend along the gradient of potential *U*_*g*_ to find a location with weaker repulsion. The continuous repulsion can account for post-meristematic events such as the mechanical stress on epidermal cells caused by the enlargement of primordia [63, 64] or the gene expression that regulates the primordial boundary [42]. The present formulation (Eq 3) is similar to the contact pressure model, which has been proposed for re-correcting the divergence angle after initiation [25, 45, 46]. Another type of post-initiation angular rearrangement has been modeled as a function of the primordia age employed as *i* −*j* −1 in the present model (Eq 2) and the distance between primordia with some stochasticity [65]. Eq 3 accounts for not only the angular rearrangement but also the radial rearrangement with stochasticity in both directions as will be described in the next subsection.

### Numerical experiments

We modeled the initiation process numerically by calculating the potential *U*_*ini*_ (Eq 2) for angular position *θ* incremented by 0.1 degree on the edge of the circular meristem. We introduced a new primordium at the position where the value of *U*_*ini*_ took the minimum, provided that the first primordium is initiated at *θ* = 0. We modeled the growth process by using a Monte Carlo method [66] to calculate the movement of primordia in the outside of the meristem depending on the potential *U*_*g*, *k*_ (Eq 3, Fig 2C). After the introduction of a new primordium, we randomly chose one primordium indexed by *k* from among the existing primordia and virtually moved its position (*r*_*k*_, *θ*_*k*_) to a new position $\left(r_{k}^{\prime },\theta_{k}^{\prime }\right)$ in the outer meristem $\left(r_{k},r_{k}^{\prime }\geq R_{0}\right)$. The new radius $r_{k}^{\prime }$ and the angle $\theta_{k}^{\prime }$ were chosen randomly following a two-dimensional Gaussian distribution whose mean and standard deviation were given by the previous position (*r*_*k*_, *θ*_*k*_) and by two independent parameters, (*σ*_*r*_, *σ*_*θ*_/*r*_*k*_), respectively. Whether or not the *k*-th primordium moved to the new position was determined by the Metropolis algorithm [66]; the primordium moved if the growth potential (Eq 3) of the new position was lower than that of the previous position (i.e., $U_{g,k}(r_{k}^{\prime },\theta_{k}^{\prime })<U_{g,k}(r_{k},\theta_{k})$). Otherwise, it moved with the probability given by

$$\begin{array}{c} P_{MP}=\exp(-\beta \Delta U_{g}), \end{array}$$ (4)

where $\Delta U_{g}=U_{g,k}(r_{k}^{\prime },\theta_{k}^{\prime })-U_{g,k}(r_{k},\theta_{k})$ and *β* is a parameter for stochasticity. This stochasticity represents a random walk biased by the repulsion potential. A case *P*_*MP*_ = 0 represents that primordia movement always follows the potential (Δ*U*_*g*_ < 0). The first primordium stays at the meristem edge *r* = *R*_0_ until the second one arises when *P*_*MP*_ = 0 because the growth potential is absent, while it can move randomly outside of the meristem when *P*_*MP*_ ≠ 0. To maintain the physical time interval of the initiation process at *τ* steps for each primordium, the number of iteration steps in the Monte Carlo simulation during each initiation interval was set to *iτ*, where *i* denotes the number of the primordia. We also studied the movement following *U*_*g*_ by numerical integration (fourth-order Runge-Kutta method) of ordinary differential equations to confirm the independence of the numerical methods (S1 Fig). All our programs were written in the C programming language and used the Mersenne Twister pseudo-random number generator (http://www.math.sci.hiroshima-u.ac.jp/m-mat/MT/emt.html) [67].

Because the initiation time interval is constant, one possible scenario for forming a whorled pattern should involve decreasing or arresting the radial displacement of primordia (Fig 1, forth row). Therefore, we focused on the change in radial position and velocity to find the whorled arrangement, while angular positions were not taken into account in the present manuscript.

## Results/Discussion

### Mutually repulsive growth promotes a whorled arrangement from sequential initiation at the proper meristem size

Numerical simulations showed that several whorls self-organized following the sequential initiation of primordia. Although several previous phyllotaxis models showed the transition between a spiral arrangement following sequential initiation and a whorled arrangement following simultaneous initiation [15, 16, 19], they were not able to reproduce the emergence of a whorled arrangement following sequential initiation, which is the situation observed in many eudicot flowers (Fig 1) [34, 37, 38, 40, 41]. In the present model, a tetramerous whorl appeared spontaneously that exhibited four primordia almost equidistant from the meristem center (Fig 2D, left and middle), by arresting radial movement of the fifth primordium at the meristem edge until the seventh primordium arose (arrowhead in Fig 2D, right). Likewise, subsequent primordia produced the same gap in radial distance for every four primordia (Fig 2D, middle and right), leading to several whorls comprising an identical number of primordia (Fig 2D). The radial positions of all primordia were highly reproducible despite stochasticity in the growth process (error bars in Fig 2D–2F, middle and right). Therefore, we identified the whorled arrangement by radial displacement arrest (arrowhead in Fig 2D, right).

The initiation order and angle of the first tetramerous whorl in the model reproduced those observed in *A*. *thaliana* sepals [68] (S2A Fig). The first primordium scarcely moved from the initiation point until the second primordium arose because growth repulsion was absent. The second primordium arose opposite the first, whereas the third and fourth primordia arose perpendicular to the preceding two. The angular position of the primordia did not change once the whorl was established because the primordia within a whorl blocked the angular displacement by the growth potential *U*_*g*_ (S3 Fig).

Introducing mutual repulsion among the primordia throughout the growth process caused the whorled arrangement to spontaneously emerge (Fig 2D). This was in contrast to the model of constant growth in which all primordia move away depending only on the distance from the floral apex [49]. A study of post-meristematic regulation by the organ-boundary gene *CUP-SHAPED COTYLEDON2* (*CUC2*) showed that *A*. *thaliana* plants up-regulating *CUC2* gene have an enlarged primordial margin and have whorled-like phyllotaxis following the normal helical initiation of primordia [42], suggesting that repulsive interactions among primordia after initiation are responsible for the formation of the floral whorls.

In the present model, the meristem size *R*_0_ controls the transition from non-whorled (Fig 2E) to whorled arrangement (Fig 2D). Radial spacing of the primordia was regular when *R*_0_ was small (Fig 2E, middle) because the older primordia pushed any new primordium across the meristem (Fig 2E, left), causing continuous movement at the same rate (Fig 2E, right). Above a threshold meristem size *R*_0_, a tetramerous whorl appeared spontaneously. The primordium number within each whorl increased up to eight with increasing *R*_0_, but the number tended to be more variable (S2B Fig). In the *A*. *thaliana* mutant *wuschel*, which has a decreased meristem size, the pattern of four sepals does not have square positions at the stage when the wild-type plant forms a tetramerous sepal whorl [69]. Conversely, the *clavata* mutant, which has an increased meristem size, has excessive floral organs with larger variation [69]. Our model consistently reproduced not only the transition from the non-whorled arrangement (Fig 2E) to the tetramerous whorled arrangement (Fig 2D) but also the variable increase in the primordia number within a whorl as the meristem size *R*_0_ increased.

The pentamerous whorl stably appeared in the presence of temporal decay of initiation inhibition (α > 0 in Eq 2). The whorls comprising five primordia appeared in the same manner as the tetramerous whorls, namely, via the locking of the sixth primordium at the initiation site (Fig 2F, right; S2C Fig).

### Developmental preference for particular organ number within a whorl

**Fig 3:**
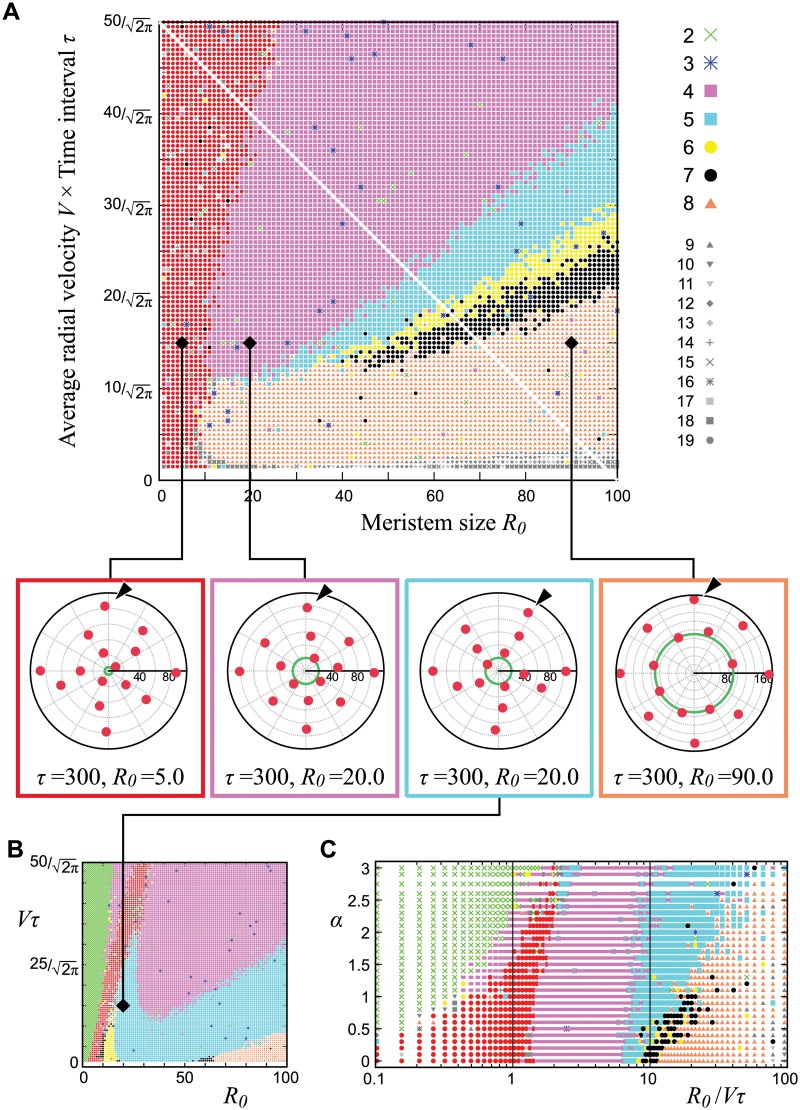
Merosity of the first whorl. **A, B.** The number of primordia before the first arrest (arrowheads in Fig 2C and 2E) is depicted by colors in the legend. The red region indicates a non-whorled pattern. For simplicity, we set *P*_*MP*_ = 0 (Eq 4) so that primordia could not move against the potential gradient *U*_*g*, *k*_. *λ*_*ini*_ = *λ*_*g*_ = 10.0, *σ*_*r*_ = *σ*_*θ*_ = 0.05. *α* = 0.0 (**A**) and *α* = 2.0 (**B**). The four panels between **A** and **B** are representative examples of each merosity where the arrowhead indicates the third primordium. **C.** Phase diagram of the first-whorl merosity according to *α* and *R*_0_/*Vτ* at $V\tau =(-0.5R_{0}+50)/\sqrt{2\pi }$ (white line in **A**). The color code is the same as that in **A** and **B**. The region of dimerous arrangement (green) increases as *α* increases, because the previous primordium becomes the most dominant inhibitor so that the new primordium initiates just opposite to the previous one and its growth is arrested by the second previous one.

In order to study the organ number within each whorl extensively, known as the merosity [70], we counted the number of primordia existing prior to the arrest of primordium displacement, which corresponds to the merosity of the first whorl (arrowheads in Fig 2D and 2F, right). We defined arrest of primordium displacement as occurring when the ratio of the initial radial velocity of a new primordium immediately after initiation to that of the previous primordium was lower than 0.2. The definition does not affect the following results as long as the ratio is between 0.1 and 0.6. We found that the key parameter for merosity is the relative value of *R*_0_ normalized by the average radial velocity $V=\sigma_{r}/\sqrt{2\pi }$ (see S1 Text) and the initiation time interval *τ* (Fig 3). The arrest of radial displacement did not occur below a threshold of *R*_0_/*Vτ* (the left region colored red in Fig 3A), whereas the whorled arrangement appeared above the threshold value of *R*_0_/*Vτ*. As *R*_0_/*Vτ* increased further, tetramery, pentamery, hexamery, heptamery, and octamery appeared, successively (Fig 3A).

The present model showed dominance of special merosity, i.e., tetramery and octamery in the absence of temporal decay of inhibition (*α* = 0 in Eq 2; Fig 3A); pentamery in the presence of temporal decay (*α* > 0; Fig 3B and 3C), in contrast to previous phyllotaxis models for whorled arrangement in which the parameter region leading to each level of merosity decreased monotonically with increasing merosity [19]. The major difference between *α* = 0 and *α* > 0 was that *θ*_3_, the angular position of the third primordium, took an average value of 90 degrees when *α* = 0 (arrowhead in Fig 3A bottom magenta panel) and decreased significantly as *α* increased (arrowhead in Fig 3A bottom cyan panel). In a pentamerous flower *Silene coeli-rosa*, the third primordium is located closer to the first primordium than the second one [34]. This is consistent with the third primordium position at *α* > 0, indicating the necessity of *α*, as we will discuss in the next section. The parameter region *R*_0_/*Vτ* for pentamery expanded with increasing *α*, whereas the border between the whorled and non-whorled arrangements was weakly dependent on *α* (Fig 3C). The tetramery, pentamery, and octamery arrangements were more robust to *R*_0_/*Vτ* and *α* than the hexamery and heptamery arrangements. Dominance of the particular number also appears in the ray-florets within a head inflorescence of Asteraceae [71], in which radial positions show the whorled-type arrangement [72]. Meanwhile, the leaf number in a single vegetative pseudo-whorl transits between two to six by hormonal control without any preference [73].

Moreover, the transition between the different merosities occurred directly, without the transient appearance of the non-whorled arrangement. This is in contrast to an earlier model [19] in which the transition between different merosity always involved transient spiral phyllotaxis. The fact that the merosity can change while keeping its whorled nature in flowers (e.g., the flowers of *Trientalis europaea*[74]) supports our results. To our knowledge, ours is the first model showing direct transitions between whorled patterns with different merosities as well as preferences for tetramery and pentamery, the most common merosities in eudicot flowers.

### Reconstructing the *Silene coeli-rosa* pentamerous whorl arrangement

**Fig 4:**
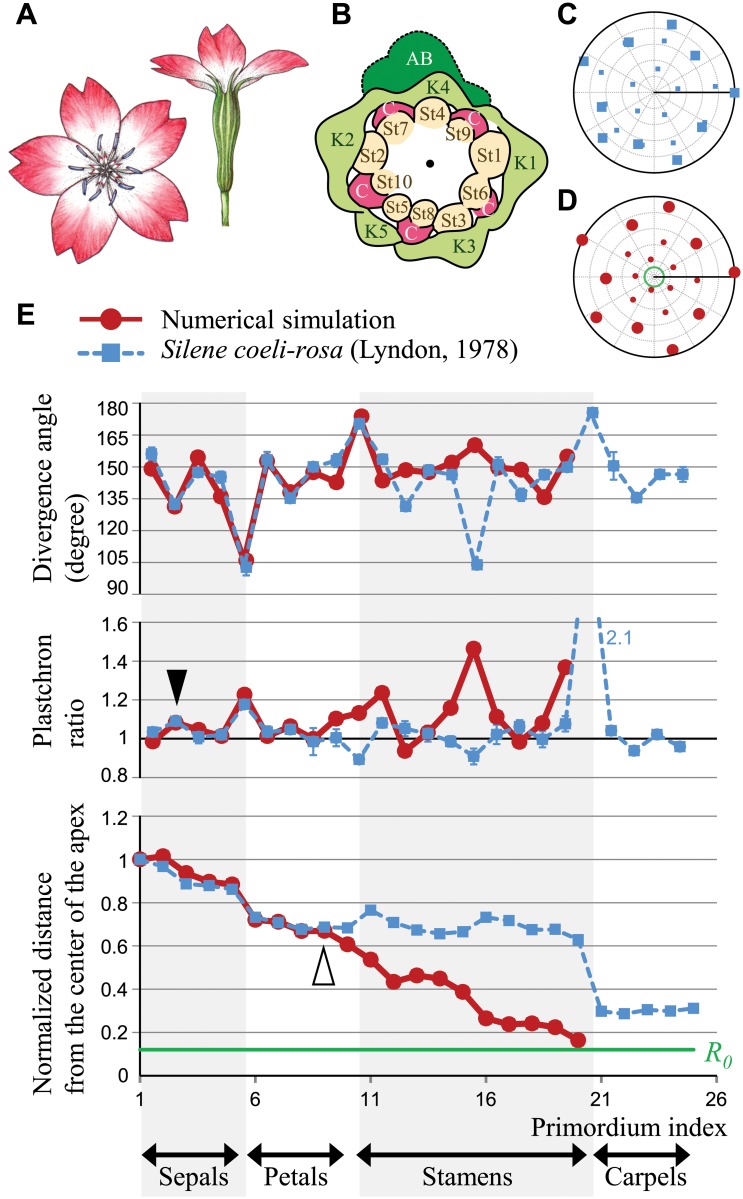
Reconstructing pentamerous floral development. **A.** Flower of *Silene coeli-rosa* (Caryophyllaceae). **B.** Reproduction of the *S*. *coeli-rosa* floral meristem traced from an SEM image by Lyndon [34]; the colors were modified. Numbers indicate the initiation order. K (sepals), C (petals), St (stamens), AB (axillary bud). **C.** Average position of the *S*. *coeli-rosa* floral primordia reconstructed from the divergence angle and plastochron ratio (**E**) measured by Lyndon (Table 1 in [34]). The number of measured apices is 9 for sepals, 5 for petals, 7 for stamens, and 2 for carpels. The positions of sepals and petals are depicted in large squares, and those of stamens and carpels are depicted in small squares. **D.** Spatial pattern of the model simulation. The first ten primordia are shown by large circles, and the subsequent ten primordia are shown by small circles. *τ* = 600, *R*_0_ = 30.0, *α* = 3.0, *σ*_*r*_ = 0.05, *σ*_*θ*_ = 5.0, *λ*_*ini*_ = *λ*_*g*_ = 20.0, *P*_*MP*_ = 0. **E.** Divergence angle (top panel) and plastochron ratio (middle) between two succeeding primordia, and the distance from the center of the apex (bottom panel) in *S*. *coeli-rosa* (blue squares) and in the model simulation (red circles). The order of petal initiation was estimated from that of the adjacent stamens (St6-St10 in **B**) following the experimental report [34]. The measurements agree with the model until the ninth primordium (open arrowhead). Error bars for the divergence angle and plastochron ratio of *S*. *coeli-rosa* denote the standard errors. Because the absolute values of the *S*. *coeli-rosa* primordia radii were not published, the distance from the center is normalized by the radius of the first sepal. The values of the parameters are the same as those in **D**. The green line (**D** and **E** bottom panel) indicates the meristem boundary in the simulation.

To further validate our model of the pentamerous whorl arrangement, we quantitatively compared its results with the radial distances and divergence angles in eudicot flowers. Here we focus on a Scanning Electron Microscope (SEM) image of the floral meristem of *S*. *coeli-rosa*, Caryophyllaceae (Fig 4A–4C) [34], because *S*. *coeli-rosa* exhibits not only five sepals and five petals in alternate positions, which is the most common arrangement in eudicots, but also the helical initiation of these primordia, which we targeted in the present model. In addition, to our knowledge, this report by Lyndon is the only publication showing a developmental sequence for both the divergence angle Δ*θ*_*k*, *k*+1_ = *θ*_*k*+1_ −*θ*_*k*_ (0 ≤ Δ*θ*_*k*, *k*+1_ < 360) and the ratio of the radial position, *r*_*k*_/*r*_*k*+1_, referred to as the plastochron ratio [75], in eudicot floral organs. Reconstructing such developmental sequences of both radial and angular positions is an unprecedented theoretical challenge, while those which describe the angular position alone for the ontogeny of spiral phyllotaxis (180 degree, 90 degree and finally convergence to 137 degree [16, 33]; the ‘M-shaped’ motif, i.e., 137, 275, 225, 275 and 137 degrees [76, 77]) have been reproduced numerically.

By substituting the initial divergence angle between the first and second sepals of *S*. *coeli-rosa* into Δ*θ*_1,2_ = 156 but not any plastochron data into the simulation (*θ*_1_ = 0 and *θ*_2_ = 156 degree), we numerically calculated the positions of the subsequent organs (Fig 4D). The observed divergence angle Δ*θ*_2,3_ = 132 degree indicates *α* > 0, because Δ*θ*_2,3_ = Δ*θ*_1,3_ = (360−156)/2 = 102 degree at *α* = 0, in the present model setting *r*_1_ ≅ *r*_2_. Even when *r*_1_ > *r*_2_, the divergence angle was calculated as Δ*θ*_2,3_ = 113 degree (*r*_1_ = *R*_0_+2*Vτ*, *r*_2_ = *R*_0_+*Vτ*, *R*_0_ = 1, *Vτ* = 0.14, and *λ*_*ini*_ = 0.05 estimated from the *S*. *coeli-rosa* SEM image [34]; see S4 Fig for detail), which is still less than the observed value. As *α* became larger, the inhibition from the second primordium became stronger than that from the first one, making Δ*θ*_2,3_ consistent with the observed value in *S*. *coeli-rosa* (Fig 4E, top).

For the subsequent sepals and petals, the model faithfully reproduced the period-five oscillation of the divergence angle and the plastochron ratio until the ninth primordium (Fig 4E), notably in the deviation of the divergence angle from regular pentagon (144 degree) and the increase of plastochron ratio at the boundary between the sepal and petal whorls. Moreover, a similar increase in the plastochron ratio occurred weakly between the second and third primordia in the first whorl (closed arrowhead in Fig 4E), indicating a hierarchically whorled arrangement (i.e., whorls within a whorl). Such weak separation of the two outer primordia from the three inner ones within a whorl is consistent with the quincuncial pattern of sepal aestivation that reflects spiral initiation in many of eudicots with pentamerous flowers (e.g., Fig 2D–E in [21]). Even with an identical set of parameters, the order of initiation in the first pentamerous whorl can vary depending on the stochasticity in the growth process. The variations of the initiation order in simulations may be caused by the absence of the outer structure, because the axillary bud seems to act as a positional information for the first primordia in *S*. *coeli-rosa* floral development (Fig 4B). The positioning of the five primordia in the first whorl was reproducible in 70% of the numerical replicates, within less than 20 degrees of that in *S*. *coeli-rosa* or that of the angles in a regular pentagon. Mismatches in the inner structure (from the tenth primordium, i.e., the last primordium in petal whorl) might be due to an increase in the rate of successive primordia initiation later in development [35], which we did not assume in our model. The agreements between our model and actual *S*. *coeli-rosa* development of sepals and petals in both the angular and the radial positions suggests that the *S*. *coeli-rosa* pentamerous whorls are caused by decreasing inhibition from older primordia.

### Mechanism for the tetramerous whorl emergence

A possible mechanism to arrest the radial displacement of a new primordium, a key process for whorl formation (arrowheads in Fig 2D and 2F), involves an inward-directed gradient of the growth potential *U*_*g*, *k*_ (Eq 3) of a new primordium so that its radial movement is prevented. To confirm this for tetramerous whorl formation (Fig 3A), we analytically derived the parameter region such that the radial gradient of the growth potential at the angle of the fifth primordium *U*_*g*,5_ (Eq 3), which is determined by the positions of the preceding four primordia, is inward-directed. For ease in the analytical calculation, we set *α* = 0 and *P*_*MP*_ = 0. The first four primordia positions were intuitively estimated (see S2 Text) as

$$\begin{array}{c} \begin{array}{cc} r_{1}=R_{0}+3\tau V, & \;\theta_{1}=0\\ r_{2}=R_{0}+3\tau V, & \;\theta_{2}=180\\ r_{3}=R_{0}+2\tau V, & \;\theta_{3}=90\\ r_{4}=R_{0}+\tau V, & \;\theta_{4}=270, \end{array} \end{array}$$ (5)

which agreed with the numerical results with an error of less than several percent regardless of the parameter spaces. Hereafter we demonstrate a case *Vτ* = 6.0. The position of the fifth primordium derived from the positions of four existing primordia (Eq 5) becomes *θ*_5_ = 90 when *R*_0_ ≤ 2, whereas *θ*_5_ ∼ 135 when *R*_0_ > 2 (S5 Fig). Next, we calculated the potential for the fifth primordium in radial direction by substituting Eq 5 and the position of the fifth primordium *θ*_5_ into Eq 3. The function becomes

$$\begin{array}{c} U_{g,5}\left(r,\theta_{5}\right)=\sum_{j=1}^{4}\exp(-\frac{d_{5j}}{\lambda_{g}})=\sum_{j=1}^{4}\exp(-\frac{\sqrt{r_{j}^{2}+r^{2}-2r_{j}r\cos\left(\theta_{j}-\theta_{5}\right)}}{\lambda_{g}})\cdot \end{array}$$ (6)

**Fig 5:**
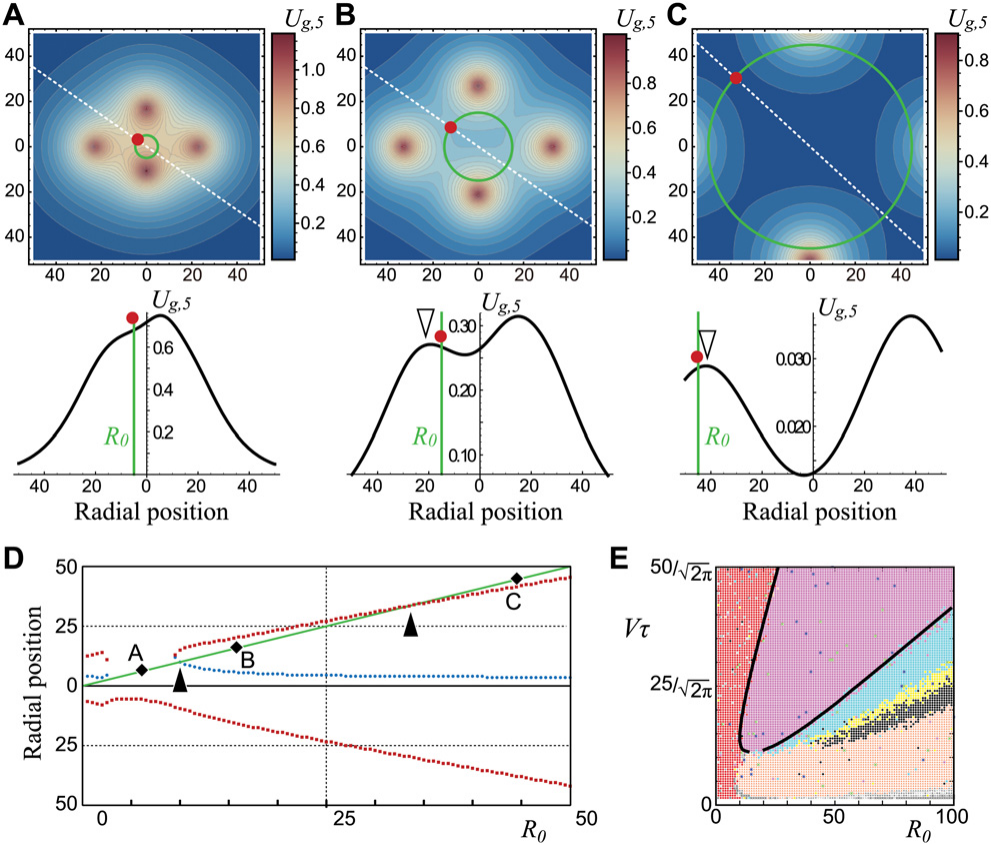
The potential landscape captures tetramerous whorl formation. **A–C.** Color-coded potential landscape (upper panel; legend) and the section (bottom panel) at the angle where *U*_*ini*_ takes the global minimum so that the fifth primordium arises (white dashed line in upper panel; Eq 6). The green line shows the meristem edge with a diameter of *R*_0_. The direction of the potential at the position where the fifth primordium arises, denoted by the red circle, is inward in **B** but outward in **A** and **C** (bottom panel). *R*_0_ = 5.0 (**A**), 15.0 (**B**), 45.0 (**C**). **D.** Radial positions that take the local minima (*r*_*min*_, blue circles) and maxima (*r*_*max*_, red squares) of potential *U*_*g*,5_ (Eq 6). Between *R*_0_ = *r*_*min*_ and *r*_*max*_, indicated by the two arrowheads, the potential at the meristem edge decreases inward as in **B**. Black diamonds correspond to the initiating position of the fifth primordium of **A**–**C**. *Vτ* = 6.0, *σ*_*θ*_ = 0.0, *λ*_*ini*_ = *λ*_*g*_ = 10.0, and *P*_*MP*_ = 0 in **A**–**D**. **E.** Superposition of the analytical result onto the numerical results (Fig 3A). Solid lines show the crossovers *r*_*min*_ = *R*_0_ and *r*_*max*_ = *R*_0_, respectively (arrowheads in **D**). *λ*_*ini*_ = *λ*_*g*_ = 10.0 and *P*_*MP*_ = 0. *σ*_*r*_ = *σ*_*θ*_ = 0.05 for numerical result, *σ*_*θ*_ = 0.0 for analytical result.

The potential exhibits a unimodal (2 < *R*_0_ < 10; Fig 5A) or bi-modal (*R*_0_ < 2, *R*_0_ > 10; Fig 5B and 5C) shape. At *R*_0_ < 10, the potential gradient at the initiation position of the fifth primordium ∂*U*_*g*,5_(*r*, *θ*_5_)/∂*r*∣_*r* = *R*_0__ is outward-directed (Fig 5A), providing almost constant growth resulting a non-whorled arrangement in the simulations (Fig 3A, red region). At *R*_0_ > 10, we defined the radial position of the local maximum closest to the fifth primordium as *r*_*max*_ (open arrowhead in Fig 5B and 5C; red squares in the upper half of Fig 5D) and the local minimum as *r*_*min*_ (blue circles in Fig 5D; 0 < *r*_*min*_ < *r*_*max*_). The potential gradient ∂*U*_*g*,5_(*r*, *θ*_5_)/∂*r*∣_*r* = *R*_0__ has a negative value when *R*_0_ < *r*_*min*_ or *r*_*max*_ < *R*_0_ (Fig 5C), causing the fifth primordium to constantly move outward. On the other hand, the potential gradient is positive, i.e., directed inward (Fig 5B), when *r*_*min*_ < *R*_0_ < *r*_*max*_ (between the two solid arrowheads in Fig 5D), causing the arrest of radial movement of the fifth primordium. The values of *r*_*min*_ and *r*_*max*_, analytically calculated as function of *R*_0_ and *τ* (solid black line in Fig 5E), were faithfully consistent with the parameter boundaries between the non-whorled pattern and the tetramerous-whorled pattern and between the tetramerous-whorled and pentamerous-whorled patterns, respectively, in the numerical simulations (Fig 5E). The assumption *r*_1_ = *r*_2_ (Eq 5) according to our numerical results (Fig 2D), which is a similar setup to co-initiation of two primordia, is not a necessary condition for consistency (S6 Fig). Thus the inward-directed gradient of the growth potential (Eq 3), which works as a barrier to arrest the outward displacement of the fifth primordium, causes the formation of tetramerous whorl.

### Mechanism for the pentamerous whorl emergence

**Fig 6:**
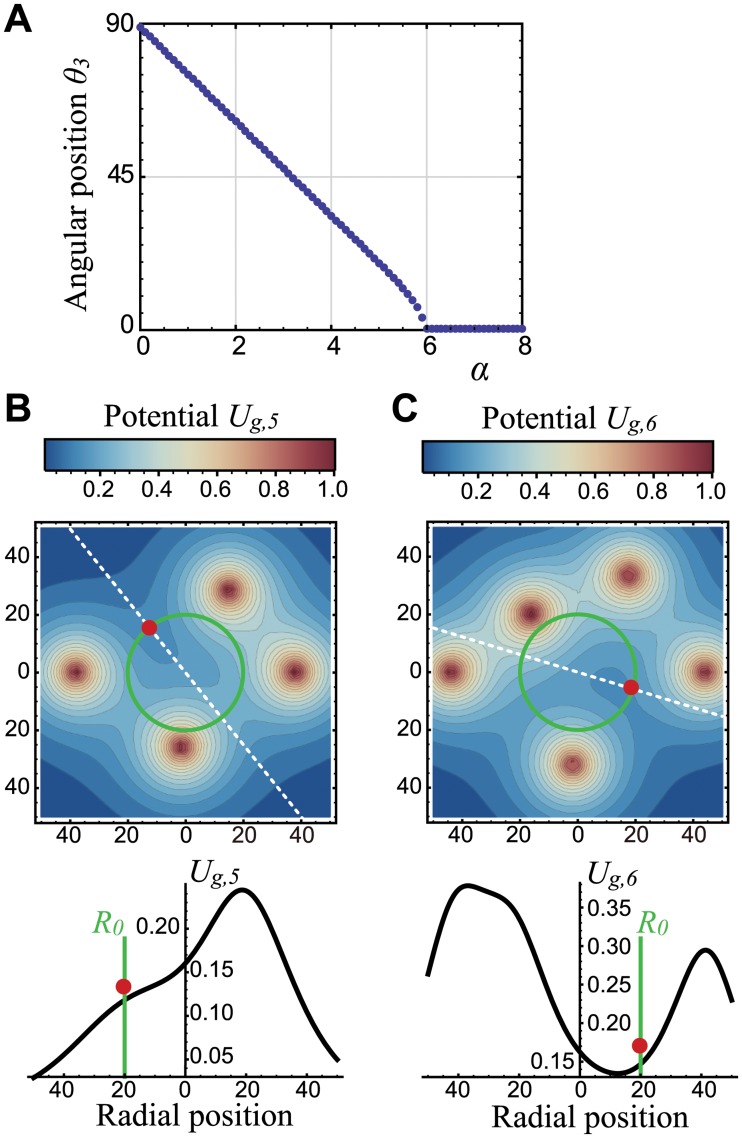
The potential landscape captures pentamerous whorl formation. **A.** The angular position of the third primordium as a function of *α*. **B–C.** Color-coded growth-potential landscape (top) and the section (bottom) at the angle where the fifth (**B**) and the sixth (**C**) primordia arise (white dashed line in the top panel). *α* = 2.0, *Vτ* = 6.0, *R*_0_ = 20.0, *σ*_*θ*_ = 0.0, *λ*_*ini*_ = *λ*_*g*_ = 10.0, and *P*_*MP*_ = 0 in **A**–**C**.

The inward radial gradient of the potential *U*_*g*, *k*_ (Eq 3) also accounted for the emergence of pentamerous whorls at *α* > 0. Unlike the case of *α* = 0, the angular position of the third primordium *θ*_3_ at the global minimum of *U*_*ini*_ decreases from 90 degrees as *α* increases (Fig 6A). For example, the recursive calculations for the minimum of *U*_*ini*_ gave the angular positions of the two subsequent primordia, *θ*_3_ ≅ 62 and *θ*_4_ ≅ 267, respectively, at *α* = 2.0 (*Vτ* = 6.0, *R*_0_ = 20.0, and *P*_*MP*_ = 0). Those angular positions were consistent with the numerical results (e.g., Fig 2F and S2B Fig). The gradient of the growth potential ∂*U*_*g*,5_(*r*, *θ*_5_)/∂*r* at the edge of the meristem for the fifth primordium that arises at *θ*_5_ ≅ 129 is negative (Fig 6B). Therefore, the fifth primordium moves outward at constant velocity so that the tetramerous whorl is unlikely to emerge. The inward-directed potential at the position of the new primordium first appears when the sixth primordium arises around 343 degrees, which was derived by the recursive calculation (Fig 6C). The first primordium (the rightmost potential peak in Fig 6C) prevents the outward movement of the sixth primordium (red circle in Fig 6C). Arrest of radial displacement of the sixth primordium is maintained until the seventh primordium arises to allow the radial gap between these primordia to appear (i.e., a pentamerous whorl emerged). After the appearance, the growth potential gradients of the sixth and the seventh primordia become outward-directed, providing their constant growth with keeping the radial gap to the first whorl. Likewise, the other merosities can be explained by similar recursive calculations of the angular position from the initiation potential (Eq 2) and the radial gradient of the growth potential (Eq 3).

### Relevance of the whorled arrangement to the phyllotactic parameter *G*

**Fig 7:**
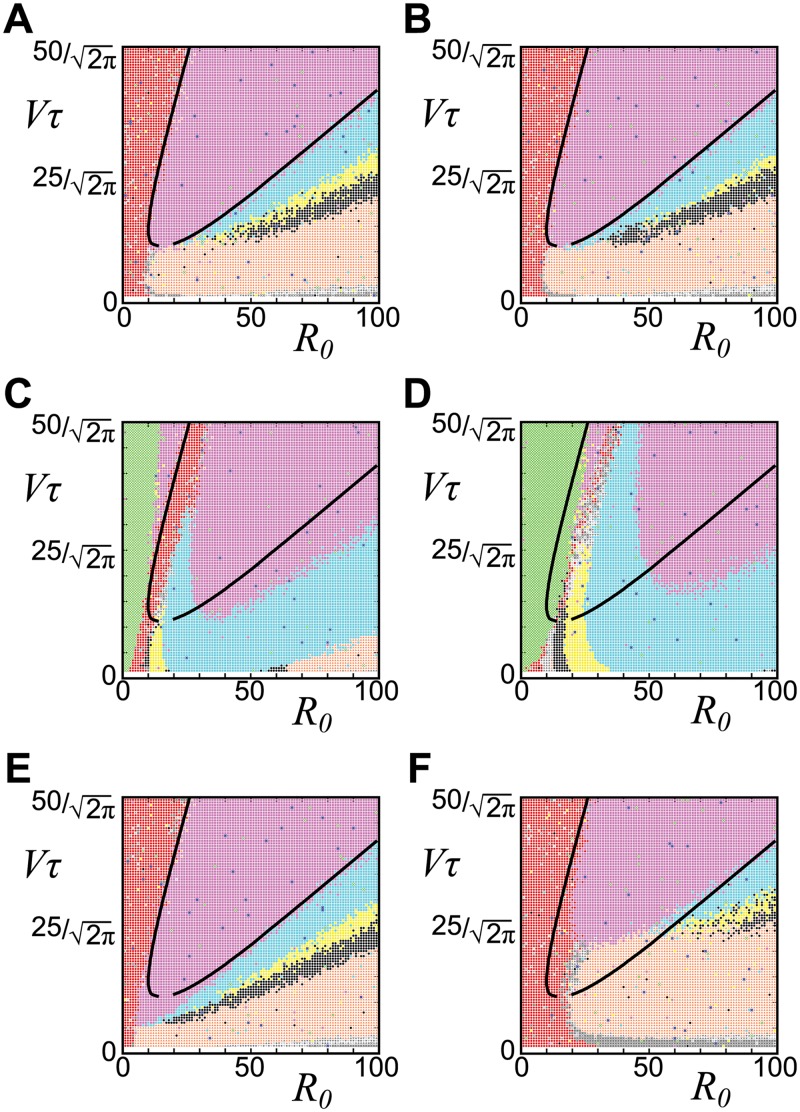
Effects of *λ*_*ini*_, *λ*_*g*_, and *α* on merosities. Superposition of the analytical result (the solid lines are identical to Fig 5E: *λ*_*ini*_ = *λ*_*g*_ = 10.0, *α* = 0.0, *P*_*MP*_ = 0) and the numerical result (*σ*_*r*_ = *σ*_*θ*_ = 0.05; the following parameters are different from the solid line: **A.** *λ*_*ini*_ = 5.0, **B.** *λ*_*ini*_ = 20.0, **C.** *α* = 2.0 **D.** *α* = 2.0, *λ*_*ini*_ = 20.0, **E.** *λ*_*g*_ = 5.0, **F.** *λ*_*g*_ = 20.0). The colors follow Fig 3. *λ*_*g*_ and *α* affect the boundary lines between each whorl as well as that between non-whorls and tetramerous whorls (**C**, **E** and **F**), whereas *λ*_*ini*_ hardly affects at *α* = 0 (**A** and **B**). At *α* ≠ 0, *α* and *λ*_*ini*_ synergistically affect the phase boundaries (**C** and **D**).

Based on these analytical results (Figs 5 and 6) and the dimensionless parameter *G* = *τV*/*R*_0_, which represents the natural logarithm of the average plastochron ratio [49, 75], we quantitatively compared the present model against previous phyllotaxis models assuming simultaneous initiation based on the initiation potential [19]. The tetramerous and pentamerous whorls appeared in, at most, 1.3-fold and 1.2-fold ranges of *G*, respectively, in the earlier study (Fig 4D in [19]); however, they appeared in much wider ranges in our model (i.e., 3-fold to 5-fold and 1.2-fold to 5-fold ranges of *G*, respectively; Fig 3C). Here, another key parameter is the temporal decay rate of the initiation inhibition *α* that shorten the transient process approaching to the golden angle (Fig 6A) than those of spiral phyllotaxis [32, 33]. *λ*_*ini*_, representing the gradient of the initiation potential (Eq 2), little affected the border between the whorled and non-whorled arrangements at *α* = 0 (Fig 7A and 7B); *λ*_*ini*_ affected the border only when *α* ≠ 0 (Fig 7C and 7D). The independency of *λ*_*ini*_ at *α* = 0 is consistent with the result shown by the previous model, which did not incorporate temporal decay of the potential and indicated that the phyllotactic pattern depends little on the functional type of initiation potential [49]. On the other hand, the gradient of the growth potential (Eq 3) regulated by *λ*_*g*_ caused a drastic transition between the whorled and non-whorled arrangements (Fig 7E and 7F). Unlike *G*, *λ*_*ini*_, and *α* (Fig 6A), *λ*_*g*_ hardly affects the angular position, as demonstrated in the previous sections, but it controls how far the growth potential works as a barrier to determine the merosities of the whorls (Fig 7E and 7F). Thus, *λ*_*g*_, *α*, and *G* differentially regulate phyllotaxis of the floral organs, suggesting the involvement of distinct molecular or physiological underpinnings.

### Predictions

We have seen that both the mutual repulsion of growth regulated by *λ*_*g*_ and the temporal decay of initiation inhibition controlled by *α* are responsible for the formation of tetramerous and pentamerous whorls following sequential initiation. These mechanisms can be experimentally verified by tuning *λ*_*g*_ and *α*. Here, we discuss several candidates for the molecular and physiological underpinnings.

#### The mutual repulsion of the growth

Because the gradient of the growth potential is the main cause of the whorl formation in our model (Figs 5 and 6), experimentally manipulating the potential decay length *λ*_*g*_ can induce the transition between the different whorl arrangements. There are two biological properties that could account for the inhibitory distance *λ*_*g*_: mechanical contact pressure between primordia and gene expression that establishes the floral organ boundary.

#### Mechanical contact pressure between primordia

Surface buckling could account for *λ*_*g*_ [29], with a wavelength regulated by mechanical properties [30] such as the expansibility of the cell wall [78].

#### Floral organ boundary establishment

Genes encoding NAM-ATAF-CUC (NAC) domain transcription factors, including *CUC1*, *CUC2*, and *No Apical Meristem* (*NAM*), are expressed at the organ boundaries and play a central role in establishing and maintaining organ boundaries [79]. In gain-of-function mutants of both *CUC1* and *CUC2*, their expression domain becomes enlarged (e.g., Fig 5C and D in [80]) and the whorled arrangement is disrupted with extra sepals and petals [80–83]. The expression breadth of *NAC* genes which establish the boundary between organs can be represented as *λ*_*g*_ in the present model. In model simulations, doubling *λ*_*g*_ consistently disrupts the tetramerous whorls, producing a non-whorled or octamerous arrangement (Fig 7F; the tetramerous region at *λ*_*g*_ = 10.0 bounded by the solid lines turns red at *λ*_*g*_ = 20.0, indicating a non-whorled arrangement, or orange, indicating an octamerous arrangement). Our model further predicts that the tetramerous arrangement will be maintained even when *λ*_*g*_ decreases by half (Fig 7E; the tetramerous region at *λ*_*g*_ = 10.0 bounded by solid lines is included in tetramerous region at *λ*_*g*_ = 5.0 denoted by the magenta points in Fig 7E), corresponding to the *A*. *thaliana* loss-of-function *cuc* mutant that shows no changes in sepal position [79]. Those consistencies suggest predictions for pentamerous flowers: weakening the post-meristematic interactions between organs will not change the merosity (the region just below the bottom solid line Fig 7E), whereas enhancing them will disrupt the whorls or increase the merosity (Fig 7F). Intriguingly, in the pentamerous flowers tomato *Solanum* *lycopersicum* and *Petunia* (Solanaceae), the role of a member of the NAC transcription factor family *NAM* seems consistent with the prediction: a *Solanum* mutant suppressing *NAM* expression exhibits fused sepals and fused whorls with keeping merosity (Fig 2 and 3 in [84]), whereas a *Petunia* *nam* mutant exhibits extra petals (Fig 3B and 4B in [85]). Further investigation of other species [86, 87] is an interesting topic for future research.

#### The temporal decay of the initiation inhibition

The temporal decay of the initiation inhibition is probably caused by the transient expression of genes in incipient primordia, which transiently increase the auxin level in the incipient primordia and decrease it in the maturing primordia. This activity decreases the involvement of older primordia in competing for auxin at the initiation site, leading to decreased initiation inhibition by the older primordia. The following two gene families of *Arabidopsis* controlling the depletion and biosynthesis of auxin exhibit transient expression and affect floral organ arrangement, thus satisfying the requirements for *α*.

#### Auxin drain into inner tissue

*NAKED PINS IN YUC MUTANTS* (*NPY*) gene families control auxin-mediated organogenesis [88, 89]. The transient expression of *NPY1* / *MACCHI-BOU 4* (*MAB4*), *NPY3*, *NPY5* in the incipient primordia in wild-type plants (Fig 2E in [90]; Fig 3A-B in [89]) indicates that auxin depletion is stronger in maturing primordia, which corresponds to *α* > 0 in the present model. In the *mab4*/*npy1* *npy3* *npy5* triple mutants, loss of PIN1 localization towards the inner tissue leads to suppressed auxin drain such that the auxin level becomes rather flat regardless of primordia age (Fig 1L-M in [59]), indicating *α* ≅ 0. Wild-type flowers, which corresponds to *α* > 0, have a tetramerous arrangement, whereas the mutants, corresponding to *α* ≅ 0, show randomized flowers, e.g., the *mab4*/*npy1* mutant possesses a disrupted tetramerous sepal whorl (Fig 1N and Table 2 in [88]), and the *npy1 npy5* double mutant exhibits more severe defects with more petals and fewer sepals (Fig 3 and Fig S2 in [89]).

#### Auxin local biosynthesis

*AINTEGUMENTA / PLETHORA* (*ANT/PLT*) genes up-regulate local auxin biosynthesis via the *YUCCA* pathway [61, 91]. The *AINTEGUMENTA-like 6* (*AIL6*)/ *PLT3* expression decreases as the primordium ages (Fig 1J in [92]; Fig 1B-C in [93]), suggesting that auxin polar transport is much weaker in the maturing floral organ primordia, as represented by *α* > 0. The *ant4 ail6* double mutant produces disrupted tetramerous whorls with a random number of floral organs (Fig 2 and Table 1 in [92]).

The dimensionless parameter *R*_0_/*Vτ* of *Arabidopsis* was estimated to be around or more than 10 (from Fig 2D in [68]). At the region, the phase diagram (Fig 3C) of the present model consistently predicts that the decrease in *α* in *Arabidopsis* caused a transition from tetramerous whorl to a fuzzy border consisting of tetramerous and pentamerous whorls, indicating that the organ number in each whorl was random. We predict that mutation in other genes with such properties (i.e., transient expression that increases the auxin level in younger primordia and decreases it in older ones) would also lead to randomized flower formation. Thus, positive *α* is a key factor for stabilizing floral organ number in *Arabidopsis*.

### Future problems

Future studies should also clarify the limits and applicability of the common developmental principle elucidated here by exploring more complex development in a wide variety of flowers. Because our model assumes sequential initiation of the primordia, it does not cover the floral development of all eudicots; sepal primordia arise simultaneously in some eudicot clades (Fig 1; e.g., mimosoid legume [94]). Likewise, in later development, several primordia arise at once in the stamen and carpel whorls (e.g., Ranunculaceae [35]). The transitions between simultaneous and sequential development have two additional intriguing implications for evolutionary developmental biology. First, the initiation types may affect the stochastic variation of floral organ numbers, possibly caused by the absence or presence of pseudo-whorls (Fig 1) and the noisy expression domain of homeotic genes [95]. Second, such transitions occur even in animal body segmentation [3, 4], possibly caused by evolution of both gene regulatory network topologies and embryonic growth [7, 9–11]. The limitations of the model can be reduced by introducing initiation whenever and wherever the potential (Eq 2) is below a threshold, allowing simultaneous as well as sequential initiation [19]. The threshold model exhibiting both types of initiation does not by itself result in the dominance of particular merosities [19]. Incorporating two mechanisms, mutual growth repulsion and temporally decreasing inhibition at the point of initiation, into the threshold model could explain the dominance of particular merosities following both the sequential and the simultaneous initiation of floral organ primordia (Fig 1). Another problem is the absence of trimerous whorls in the present model (Fig 3). The transition between the trimery and tetramery or pentamery, and vice versa, occurred multiple times during the evolution of angiosperms. Therefore, trimerous flowers are scattered across the basal angiosperms, monocots, and a few families of eudicots [96, 97]. Elucidating the developmental mechanisms underlying the transitions between the different merosities, as well as those between sequential and simultaneous initiation, will be an important avenue for future studies.

### Conclusion

One problem in determining floral organ number is how to generate whorls comprised of a specific number of organs. By introducing a growth assumption (i.e., continuous repulsion among primordia throughout development, which was originally proposed as the contact pressure model [25, 45, 46] and is supported by experimental observations [42]) into a dynamical model of phyllotaxis [49], we showed that the whorled arrangement arises spontaneously from sequential initiation. Moreover, when we allowed the inhibition to decay over time [33, 47, 48], pentamerous whorls became the dominant pattern. The merosity tended to be four or five in much larger parameter spaces than those in which it tended to be six or seven. The emergence of tetramerous and pentamerous whorls could be verified experimentally by tuning the two parameters *α* and *λ*_*g*_.

## Supporting Information

S1 Text Analytical derivation of the average radial velocity during growth. (PDF)

S2 Text Estimation of the fifth primordium position at *α* = 0. (PDF)

S1 Fig Supporting figure of Fig 2. The tetramerous (**A**) and pentamerous (**B**) pattern generated by the ordinary differential equations *dr*_*k*_/*dt* = −∂*U*_*g*, *k*_/∂*r*_*k*_, *dθ*_*k*_/*dt* = −(1/*r*_*k*_)∂*U*_*g*, *k*_/∂*θ*_*k*_, was used instead of the Monte Carlo method. We confirmed (1) the emergence of whorled arrangements as shown in this figure (consistent with Fig 2) and (2) the dominance of the tetramery at *α* = 0 and the pentamery at *α* > 0 (consistent with Fig 3). *λ*_*g*_ = *λ*_*ini*_ = 2.5. **A.** *R*_0_ = 3.0, *τ* = 17.5, *α* = 0.0. **B.** *R*_0_ = 4.5, *τ* = 25, *α* = 2.0.
(EPS)

S2 Fig Organ positioning time course. **A.** Organ positioning time course of tetramerous whorls (Fig 2D) and **B.** The increase of primordium number within a whorl with increasing *R*_0_. The left panel shows the radial distance (black) from the meristem center as a function of the primordium initiation index averaged over 400 replicate Monte Carlo simulations. Error bars represent 2 S.D. Red circles are a set of representative samples, whose spatial pattern is represented in the small panel at the top-right. Yellow circles are another set of samples. Note that the primordium number within a whorl is different between replicates. When these circles are sorted by their radii, the number within a whorl takes six in red set and five in the yellow set. Right panel: Time evolution of the radial coordinates of each primordium averaged over 400 replicates. Error bars show 2 S.D. **C.** Organ positioning time course of pentamerous whorls (Fig 2F). Green line denotes the meristem edge. (*R*_0_, *α*) = (20.0,0.0) in **A**, (25.0,0.0) in **B** and (20.0,2.0) in **C**. *β* = 1.0 × 10^4^, *λ*_*ini*_ = *λ*_*g*_ = 10.0, *τ* = 300, and *σ*_*r*_ = *σ*_*θ*_ = 0.05.
(EPS)

S3 Fig Growth potential landscape. The growth potential *U*_*g*,4_ is shown as a function of the angular position *θ* at the radius of the fourth primordium in Fig 5B. The small panel shows the landscape of potential *U*_*g*,5_ at the corresponding time (identical to the upper panel of Fig 5B). Arrowheads indicate the position of the fourth primordium.
(EPS)

S4 Fig Analytical calculation at *α* = 0 could not account for the third primordium position of *Silene coeli-rosa*. **A.** Black circles 1–3 demonstrate the positions of the first to third primordia. The angles were from experimental observation [34] and radii were estimated as *r*_1_ = *R*_0_+2*Vτ*, *r*_2_ = *R*_0_+*Vτ*, and *r*_3_ = *R*_0_. *R*_0_ = 1.0 and *Vτ* = 0.14 were obtained from the radius of the centermost carpel, which we assumed as the meristem edge and average of the radial difference between successive sepals, respectively, normalized by the radius of the centermost carpel. A red circle 3^′^ shows the position of the third primordium analytically calculated from the observed positions of the first and second ones at *α* = 0. *λ*_*ini*_ = 0.05. **B.** The divergence angle between the third and second (Δ*θ*_2,3_), as well as third and first primordia (Δ*θ*_1,3_), as a function of *λ*_*ini*_. In **A** and **B**, the observed Δ*θ*_2,3_ and Δ*θ*_1,3_ are represented in blue and pale blue, respectively, whereas the calculated Δ*θ*_2,3_ and Δ*θ*_1,3_ are depicted in red and pale red, respectively.
(EPS)

S5 Fig Supporting Figure of Fig 5. After the initiation of four primordia at the positions given by Eq 5, the angular positions that occupy the local minima (blue) and maxima (red) of potential *U*_*ini*_ at the edge of the meristem (Eq. S2) are plotted as a function of *R*_0_. Blue solid circles denote the global minima, which represent the position of the fifth primordium, whereas the blue open circles around 200 and 340 degrees at *R*_0_ > 15 signify the local minima. Solid black diamonds correspond to Fig 5A–5C.
(EPS)

S6 Fig The phase diagram when *r*_1_ > *r*_2_. The initial position of the first primordium was set to *r*_1_ = *R*_0_+*Vτ* and *θ*_1_ = 0.0. Thus, *r*_1_ ≅ *R*_0_+2*Vτ* and *r*_2_ ≅ *R*_0_+*Vτ* were yielded when the third primordium arose, whereas in the model discussed in the main text *r*_1_ = *R*_0_ and *θ*_1_ = 0.0 such that *r*_1_ ≅ *r*_2_. Parameters are the same as Fig 3A. Black solid lines show the analytical results produced when *r*_1_ = *r*_2_, which is identical to Fig 5E. Although the parametric region of the tetramery was slightly right-shifted and the border between tetramery and pentamery became fuzzy, tetrameric dominance was maintained.
(EPS)
