# Supplementary material for: A Dynamical Phyllotaxis Model to Determine Floral Organ Number
Source: PLoS Comput Biol. 2015 May 7;11(5):e1004145. doi: 10.1371/journal.pcbi.1004145 (PMC4423988; doi:10.1371/journal.pcbi.1004145)
Supplement: S1 Text — (PDF) [file pcbi.1004145.s001.pdf]

A Dynamical Phyllotaxis Model to Determine Floral Organ Number.

Miho S. Kitazawa[1,2,*], Koichi Fujimoto[1,*]

1 Department of Biological Sciences, Osaka University, Toyonaka, Osaka, Japan.
2 Japan Society for the Promotion of Science, Tokyo, Japan.

*** fujimoto@bio.sci.osaka-u.ac.jp (KF); kitazawa@bio.sci.osaka-u.ac.jp (MSK)**

S1 Text

Analytical derivation of the average radial velocity during growth. The radial velocity of primordia averaged over the growth process is approximately derived by integrating Monte Carlo steps. The radial displacement of the primordium k in a single Monte Carlo step given by $x = r'_k - r_k$ follows a Gaussian distribution (see Numerical experiments in the Model section) given by

$$\frac{1}{\sqrt{2\pi\sigma_r^2}} \exp\left(-\frac{x^2}{2\sigma_r^2}\right),$$

where the average and the standard deviation are zero ($r_k = r'_k$) and σ_r, respectively. At $P_{MP} = 0$, when the radial gradient of the growth potential $U_{g,k}$ is negative, the Metropolis method always selects outward movement (see Numerical experiments in the Model section) so that the actual movement follows a one-sided truncated Gaussian distribution ($x \geq 0$). The average radial velocity V is approximated as the expected value given by

$$V = \int_0^\infty \frac{x}{\sqrt{2\pi\sigma_r^2}} \exp\left(-\frac{x^2}{2\sigma_r^2}\right) dx = \frac{\sigma_r}{\sqrt{2\pi}}. \tag{S1}$$

Hence, during the time interval τ of primordia initiation, all primordia move radially a distance of $\tau\sigma_r/\sqrt{2\pi}$ on average.
