# Supplementary material for: A Dynamical Phyllotaxis Model to Determine Floral Organ Number
Source: PLoS Comput Biol. 2015 May 7;11(5):e1004145. doi: 10.1371/journal.pcbi.1004145 (PMC4423988; doi:10.1371/journal.pcbi.1004145)
Supplement: S2 Text — (PDF) [file pcbi.1004145.s002.pdf]

A Dynamical Phyllotaxis Model to Determine Floral Organ Number.

Miho S. Kitazawa[1,2,*], Koichi Fujimoto[1,*]

1 Department of Biological Sciences, Osaka University, Toyonaka, Osaka, Japan.
2 Japan Society for the Promotion of Science, Tokyo, Japan.

*** fujimoto@bio.sci.osaka-u.ac.jp (KF); kitazawa@bio.sci.osaka-u.ac.jp (MSK)**

S2 Text

Estimation of the fifth primordium position at $\alpha = 0$. To analytically calculate the conditions for tetramerous whorl formation (Fig. 3A), we intuitively estimated the first four primordial positions at $\alpha = 0$ and $P_{MP} = 0$. Because we set the angular position of the first primordium to zero ($\theta_1 = 0$), the second primordium arose at the opposite side of the meristem ($\theta_2 = 180$) farthest from the first primordium. The third and fourth primordia were initiated at the middle positions relative to the preceding primordia (i.e., at $\theta_3 = 90$ and $\theta_4 = 270$ degrees, respectively) at $\alpha = 0$ due to symmetric repression by the first and second primordia. Regarding the radial direction, the first primordium was not affected by any other primordia until the second primordium arose; therefore, the first primordium stayed at the meristem edge R_0 at $P_{MP} = 0$ (see the Model section). After initiation of the second primordium, the primordia repelled each other symmetrically following the growth potential (Eq. 3). Thus, the times spent for movement until the fifth primordium arose were 3τ for the first and second primordia and 2τ and τ for the third and fourth primordia, respectively. The average velocity of radial movement from the meristem edge was $V = \sigma_r/\sqrt{2\pi}$ (Eq. 7) for these four primordia after initiation of the second primordium. Thus, the positions of these four primordia (r_i, θ_i) in polar coordinates are given by Eq. 5. By substituting Eq. 5 into the function for the initiation potential Eq. 2, the function becomes

$$U_{ini}(\theta) = \sum_{j=1}^{4} \exp\left(-\frac{d_{5j}}{\lambda_{ini}}\right) = \sum_{j=1}^{4} \exp\left(-\frac{\sqrt{r_j^2 + R_0^2 - 2r_j R_0 \cos(\theta_j - \theta)}}{\lambda_{ini}}\right), \qquad \text{(S2)}$$

The angle θ_{min} taking the local minimum of the potential yielded the angular position of the fifth primordium, i.e., $\theta_5 = \theta_{min}$. The angle θ_{min} underwent a pitchfork bifurcation at $R_0 = 2$ and $\tau = 300$ and split from 90 degrees into 45 and 135 degrees (S5 Fig). At $R_0 > 2$, the minimum around ~ 135 degrees was selected instead of ~ 45 degrees as the initiating position of the fifth primordium without losing the generality because the potential was symmetrical around $\theta = 90$ at $\alpha = 0$. Thus, $\theta_5 = 90$ when $R_0 \leq 2$, whereas $\theta_5 \sim 135$ when $R_0 > 2$.
